# ICU admission following an unscheduled return visit to the pediatric emergency department within 72 hours

**DOI:** 10.1186/s12887-019-1644-y

**Published:** 2019-08-02

**Authors:** Charng-Yen Chiang, Fu-Jen Cheng, Yi-Syun Huang, Yu-Lun Chen, Kuan-Han Wu, I-Min Chiu

**Affiliations:** Department of Emergency Medicine, Kaohsiung Chang Gung Memorial Hospital, No.123, Dapi Rd. Niaosong Dist, Kaohsiung City, 83301 Taiwan, Republic of China

**Keywords:** Pediatric emergency department, Unscheduled return visit, ICU admission

## Abstract

**Introduction:**

The purpose of this study was to describe the demographic characteristics and prognosis of children admitted to the intensive care unit (ICU) after a pediatric emergency department (PED) return visit within 72 h.

**Method:**

We conducted this retrospective study from 2010 to 2016 in the PED of a tertiary medical center in Taiwan and included patients under the age of 18 years old admitted to the ICU after a PED return visit within 72 h. Clinical characteristics were collected to perform demographic analysis. Pediatric patients who were admitted to the ICU on an initial visit were also enrolled as a comparison group for outcome analysis, including mortality, ventilator use, and length of hospital stay.

**Results:**

We included a total of 136 patients in this study. Their mean age was 3.3 years old, 65.4% were male, and 36.0% had Chronic Health Condition (CHC). Disease-related return (73.5%) was by far the most common reason for return.

Compared to those admitted on an initial PED visit, clinical characteristics, including vital signs at triage and laboratory tests on return visit with ICU admission, demonstrated no significant differences. Regarding prognosis, ICU admission on return visit has a higher likelihood of ventilator use (aOR:2.117, 95%CI 1.021~4.387), but was not associated with increased mortality (aOR:0.658, 95%CI 0.150~2.882) or LOHS (OR:-1.853, 95%CI -4.045~0.339).

**Conclusion:**

Patients who were admitted to the ICU on return PED visits were associated with an increased risk of ventilator use but not mortality or LOHS compared to those admitted on an initial visit.

## Introduction

An unscheduled emergency department (ED) revisit, which is typically defined as a return visit within 72 h after being discharged from a previous ED visit, was a concept developed in the early 1990s [1–3]. Since then, such revisits have become a widely reviewed medical quality assessment tool. However, the most recent evidence has suggested that admissions following an unscheduled ED revisit are no longer an indicator of poor quality [4–7]. In 2018, Sills et al. demonstrated that a return visit to the Pediatric Emergency Department (PED) was not associated with increased ICU admission, mortality, or even hospital costs [6]. Other studies have primarily focused on adult patients, and both positive and negative results with regard to clinical outcomes following unscheduled return visits have been found.

However, rapid deterioration after being discharged from the ED and subsequent admission to the ICU on a return visit are still considered among the most serious adverse events involving ED patients [8, 9]. Furthermore, caregivers of children admitted during a second PED visit are usually more dissatisfied with the health care facility compared with their first visit, which puts greater pressure on physicians to handle critical events on return visits [10]. Previous studies have reported mortality rates of 19.9–27.5% for adult patients during ICU admission following unplanned ED revisits, with such associated factors as old age and underlying comorbidity [11–13]. To the best of our knowledge, no study on ICU admission after unscheduled ED revisits of pediatric patients has yet been published. Therefore, our study aimed to describe the demographic characteristics and clinical prognosis of children admitted to the ICU following a PED return visit within 72 h.

## Method

This retrospective study was conducted from January 1, 2010 to December 31, 2016 in the PED of a tertiary medical center in Southern Taiwan. About 30,000 PED visits are made to the hospital every year. This study was approved by the institutional review board of the Chang Gung Medical Foundation (IRB number: 101- 4490B). All records and information of both the patients and physicians were anonymous and de-identified prior to analysis.

For our study population, we included non-traumatic patients under the age of 18 years old admitted to the ICU straight from the PED after a return visit within 72 h of a previous PED discharge during the study period. Patients admitted to the ICU on an initial PED visit were collected to serve as the comparison group for clinical outcome analysis. Patients who returned to the PED after hospital admission were not included. Clinical characteristics included age, gender, vital signs at triage, and laboratory tests, all of which were collected to perform demographic analysis.

We classified patient diagnosis categories in this study based on the main diagnosis documented upon ICU discharge. Diagnosis categories were classified into the following five groups: infectious disease (e.g., sepsis, pneumonia, urinary tract infection, soft tissue infection), respiratory disease (e.g., asthma with acute exacerbation), digestive disease (e.g., ileus, obstructive jaundice and other hepatobiliary disorders), neurological disorder (e.g., seizure, intra-cranial hemorrhage), and others (e.g., diabetic ketoacidosis, heart failure, complications of acute leukemia). The primary disease that led to ICU admission was used for category classification. For example, if a patient was admitted for pneumonia, which was complicated with an asthma attack, pneumonia was considered the primary disease. This patient was then classified into the infectious disease category.

We also discussed the justification of the ED revisits using four separate dimensions established in previous studies [13, 14]: not-related, doctor-related (e.g., misdiagnosis or inadequate treatment), disease-related (e.g. disease complication or progression after first ED visit), and patient-related (e.g. discharge against medical advice).

Chronic health conditions (CHC) were taken into consideration in this study. CHC was initially defined by Feudtner et al. in 2000 as “any medical condition that can be reasonably expected to last at least 12 months (unless death intervenes) and to involve either several different organ systems or one organ system severely enough to require specialty pediatric care and probably some period of hospitalization in a tertiary care center,” [15]. In this study, we adopted this definition based on a revised version of it from a large ICU study performed in the U.S. in 2012 [16]. For example, cerebral palsy, epilepsy, asthma, diabetes mellitus, heart failure, leukemia, etc., will be considered as CHC in this study.

In addition to demographic data, patients who were admitted to the ICU on their “initial PED visit” (“initial” was used to separate the “first” PED visit from those admitted on a return visit) were collected and compared to the studied group. Clinical characteristics and prognosis, including mortality, ventilator assistance, and length of hospital stay, were all analyzed. We performed student t-test and Chi square analysis to determine the correlation factors of the patients admitted to the ICU on an initial or a return PED visit. To compare prognosis, logistic regression regarding the association of clinical outcome with return PED visit was performed after adjusting for other confounding factors.

## Results

**Fig. 1:**
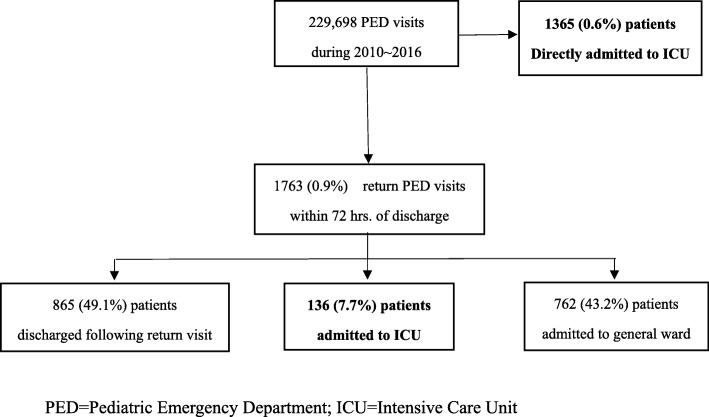
Patient inclusion flowchart in the studied hospital during the period 2010~2016. PED = Pediatric Emergency Department; ICU=Intensive Care Unit

The study period consisted of 229,698 PED visits, among which 28,012 (12.2%) patients were directly admitted, and 1365 (0.6%) patients were admitted to the ICU on their initial PED visit. Among those who were discharged from the PED on the first visit, 1763 (0.9%) patients return to the PED within 72 h, and 136 (7.7%) of them were admitted to the ICU (Fig. 1). Among patients admitted to the ICU on a return visit, 106 (77.9%) of them were younger than 6 years old, 89 (65.4%) patients were male, and 44 (32.4%) patients had a chronic health condition (Table 1). Infectious diseases, respiratory diseases, and digestive diseases accounted for 57.4, 16.9 and 12.5% of diagnoses on ICU discharge, respectively. Regarding reason for return visit, disease-related conditions (*N* = 100, 73.5%) accounted for the most common reason for revisit. Doctor-related and patient-related return visits took place for 20 (14.7%) and 11 (8.1%) patients, respectively.

**Table 1:** Demographic characteristics of patients admitted to the ICU after an unscheduled PED return visit within 3 days (*N* = 136)

Variables	Mean ± SEM / N (%)
Age (year)	3.3 ± 0.39
< 1 year old	72 (52.9)
1~3 years old	23 (16.9)
3~6 years old	11 (8.1)
6~12 years old	19 (14.0)
12~18 years old	11 (8.1)
Male	89 (65.4)
Chronic health condition	44 (32.4)
Diagnosis category on ICU discharge	
Infectious	78 (57.4)
Respiratory	23 (16.9)
Digestive	17 (12.5)
Neurological	11 (8.1)
Other	7 (5.1)
Return visit justification	
Unrelated	5 (3.7)
Doctor-related	20 (14.7)
Disease-related	100 (73.5)
Patient-related	11 (8.1)

*PED* Pediatric Emergency Department, *ICU* intensive care unit, *SEM* mean of standard deviation, *LOHS* Length of Hospital Stay

A comparison of the clinical characteristics between patients with ICU admission on initial PED visit and return visit are shown in Table 2. Compared to those admitted to the ICU on an initial PED visit, patients with ICU admission on return visits were older (3.3 ± 0.39 vs 2.2 ± 0.16 years old, *p* = 0.006), and fewer had CHC (24.5% vs. 32.4%, *p* = 0.049). Vital signs at triage, including body temperature, respiratory rate, systolic blood pressure, and diastolic blood pressure showed significant differences between both the first visit and the return visit with those admitted on the initial visit. On the other hand, we observed no statistical differences in vital signs between the first visit and return visit patient.

**Table 2:** Comparison of clinical characteristics between ICU admission on initial PED visit and admission to ICU on return visit

	ICU admission on initial PED visit	ICU admission on return PED visit within 3 days			
	Mean ± SEM / N (%)	Mean ± SEM / N (%)			*p*-value
Total	1365	136			
Age (in years)	2.2 ± 0.16	3.3 ± 0.39			0.006*
Male	829 (60.7)	89 (65.4)			0.311
Chronic Health Condition	334 (24.5)	44 (32.4)			0.049*
		First PED visit		Return PED visit	
	Mean ± SEM / N (%)	Mean ± SEM / N (%)	*p*-value	Mean ± SEM / N (%)	*p*-value
Initial Vital Signs	*N* = 1365	N = 136		N = 136	
BT (°*C*)	37.7 ± 0.03	37.4 ± 0.09	**0.003**	37.4 ± 0.10	**0.003**
Heart Rate (/min)	145 ± 0.82	141 ± 2.7	0.161	145 ± 2.6	0.895
RR (/min)	27 ± 0.2	25 ± 0.4	**0.008**	26 ± 0.4	0.754
SBP (mmHg)	98 ± 0.9	107 ± 2.6	**0.003**	106 ± 3.3	**0.007**
DBP (mmHg)	60 ± 0.7	66 ± 2.2	**0.030**	65 ± 2.5	**0.033**
Laboratory Tests	*N* = 1313	*N* = 32		*N* = 128	
WBC (k/uL)	13.2 ± 0.53	10.1 ± 0.87	0.367	13.3 ± 1.82	0.987
Creatinine	0.52 ± 0.045	0.60 ± 0.105	0.841	0.46 ± 0.154	0.633
Sugar	122 ± 0.9	121 ± 13.4	0.988	136 ± 9.6	0.279
CRP (mg/L)^a^	35.3 ± 1.92	4.0 ± 1.53	**< 0.001**	40.3 ± 6.98	0.471

*BT* body temperature, *RR* respiratory rate, *SBP* systolic blood pressure, *DBP* diastolic blood pressure, *WBC* white blood cell, *CRP* C-reactive Protein

^a^ CRP was the only parameter to show a significant difference between the first and return PED visits among patients admitted to the ICU on a return visit (*p* < 0.001)

*Patients admitted to ICU on return visit were older and more were with chronic health condition

As for laboratory tests, levels of white blood cell (WBC), creatinine, blood sugar, and C-reactive protein (CRP) were considered. Among all return visit patients, laboratory tests were obtained for 32 on the first visit. CRP levels of the first PED visit were the only significant differences found in the lab tests, being lower than those who were admitted to the ICU on the initial visit (4.0 ± 1.53 vs. 35.3 ± 1.92, *p* < 0.001). Furthermore, in the group comparison of patients admitted to the ICU on a return visit, CRP levels were lower at the first visit compared to the return visit (4.0 ± 1.53 vs. 40.3 ± 6.98, p < 0.001) (Table 2).

Since age was a significant confounding factor of vital signs in pediatric patients, we further performed stratified analysis according the different age groups (Table 3). Beside body temperature, which was found to be lower in return PED visit patients (37.3 ± 0.11 vs. 37.8 ± 0.04 °*C*, *p* < 0.001) in the infant period (age <  1-year-old), no significant differences were found among the other vital signs between the two populations.

**Table 3:** Stratified analysis of vital signs according to different age groups between ICU admission on initial PED visit and return PED visit

Age	ICU admission on initial PED visit	ICU admission on return PED visit	
	Mean ± SEM / N (%)	Mean ± SEM / N (%)	*p*-value
< 1 year	N = 948	*N* = 72	
BT (°C)	37.8 ± 0.04	37.3 ± 0.11	< 0.001*
Heart Rate (/min)	152 ± 1.3	154 ± 2.8	0.581
RR (/min)	28 ± 0.2	28 ± 0.6	0.643
SBP (mmHg)	90 ± 1.0	92 ± 4.7	0.59
DBP (mmHg)	55 ± 0.8	56 ± 3.3	0.774
1~3 years old	*N* = 114	*N* = 23	
BT (°C)	37.6 ± 0.12	37.9 ± 0.33	0.335
Heart Rate (/min)	147 ± 3.0	155 ± 5.6	0.237
RR (/min)	26 ± 0.6	27 ± 0.9	0.53
SBP (mmHg)	108 ± 2.1	120 ± 8.6	0.148
DBP (mmHg)	68 ± 1.6	74 ± 4.5	0.194
3~6 years old	*N* = 97	N = 12	
BT (°C)	37.7 ± 0.12	37.6 ± 0.29	0.81
Heart Rate (/min)	137 ± 3.0	143 ± 5.5	0.424
RR (/min)	24 ± 0.3	25 ± 0.9	0.251
SBP (mmHg)	113 ± 2.5	118 ± 6.6	0.52
DBP (mmHg)	73 ± 1.7	85 ± 8.1	0.17
6~12 years old	126	19	
BT (°C)	37.5 ± 0.12	37.4 ± 0.32	0.955
Heart Rate (/min)	128 ± 2.2	120 ± 10.4	0.273
RR (/min)	24 ± 0.4	24 ± 1.3	0.681
SBP (mmHg)	118 ± 2.6	107 ± 7.6	0.199
DBP (mmHg)	76 ± 2.0	68 ± 5.5	0.253
12~18 years old	77	11	
BT (°C)	37.3 ± 0.14	36.6 ± 0.22	0.058
Heart Rate (/min)	114 ± 3.4	95 ± 0.6.6	0.057
RR (/min)	22 ± 0.4	21 ± 1.0	0.655
SBP (mmHg)	122 ± 3.4	120 ± 9.5	0.854
DBP (mmHg)	73 ± 2.3	61 ± 7.3	0.099

*BT* body temperature, *RR* respiratory rate, *SBP* systolic blood pressure, *DBP* diastolic blood pressure

*In the group of age < 1 year old, body temperature was lower in patients with ICU admission on return visit

While comparing ICU admission on return PED visits to those admitted on initial visits (Table 4), we found higher ventilator assistance rates in the return visit group (7.4% vs. 3.2%, *p* = 0.017). In contrast, mortality (1.5% vs. 1.8%, *p* = 0.576) and LOHS (8.3 ± 0.60 vs. 9.7 ± 0.35 days, *p* = 0.207) showed no significant differences between the two groups. To control potential confounding factors, we applied logistic regression with selected prognosis as reference categories and adjusted for patients’ age, gender, and chronic health conditions (Table 5). Compared to ICU admission on initial PED visit, patients admitted to the ICU on return visits had a higher likelihood of ventilator use [aOR: 2.117 (95% CI:1.021~4.387)] but were not associated with mortality or LOHS differences. On the other hand, CHC [OR: 3.067 (95% CI: 1.536~4.598)] was associated with increased LOHS.

**Table 4:** Comparison of clinical outcomes between ICU admission on initial PED visit and return PED visit

	ICU admission on initial PED visit	ICU admission on return PED visit	
	Mean ± SEM / N (%)	Mean ± SEM / N (%)	*p*-value
Total	1365	136	
Mortality	24 (1.8)	2 (1.5)	0.576
Ventilator use	43 (3.2)	10 (7.4)	0.017*
LOHS (days)	9.7 ± 0.35	8.3 ± 0.60	0.207

*ICU* intensive care unit, *LOHS* length of hospital stay

*ICU admission on return visit was associated with increased ventilator use

**Table 5:** Regression analysis of clinical outcomes adjusting for age and gender

	ICU admission on return PED visit	Chronic health condition
	aOR (95%CI)	aOR (95%CI)
Mortality	0.658 (0.150~2.882)	1.221 (0.526~2.835)
Ventilator Assistance	2.117 (1.021~4.387)*	1.433 (0.784~2.619)
LOHS	-1.853 (−4.045~0.339)	3.067 (1.536~4.598)**

*LOHS* Length of Hospital stay, *aOR* adjusted Odds Ratio, *95% CI* 95% confidence interval

*ICU admission on return visit was associated with increased ventilator assistance

**Chronic health condition was associated with increased LOHS

## Discussion

Few studies have focused on pediatric patients admitted to the ICU after an unscheduled ED revisit. In this seven-year retrospective study, the majority of ICU-admitted patients were ultimately discharged smoothly. We reviewed and analyzed both their demographic characteristics and clinical outcomes and compared them with those admitted to the ICU on initial visits. A previous nationwide-based study in the U.S. from 2012 showed that 698,000 pediatric ED revisits (2.7%) were documented over 7 years [17, 18]. In that study, among all PED revisit patients, the ICU admission rate was about 16.7 per 100,000 PED discharges. Compared to the previous study, the return PED visit rate (0.9%) was relatively lower in our study, but with a higher ICU admission rate (7.7%) among these return visit patients.

Patients who were admitted to the ICU on initial visits were younger compared with those admitted on return visits in this study (Table 2), which likely occurred due to the infant population (*N* = 948, 69.5%) being much higher in patients admitted to the ICU on their initial PED visit. This difference in age between initial and return visit patients may explain the higher rate of CHC return visit patients, as initial visit patients tended to be younger, healthy children.

The return visit diagnoses of patients in our study were similar to those found the literature. In 2013, Easter et al. demonstrated that gastrointestinal, infectious, respiratory, and neurology diseases accounted for more than 80% of return PED visits [19]. In the same study, disease-related returns were the most common justification for return visits (72%), followed by doctor-related returns (11%). This finding also correlated with our study regarding justification of return visit. The composition of return visit justifications resembled that of another study on unplanned hospital admission within 3 days of ED discharge in adult patients, in which disease related etiology (72.0%) accounted for the majority of reasons, followed by inadequate diagnosis or management (12.2%) [12].

Patients admitted to the ICU on initial PED visits were compared to those admitted to the ICU on return visits as a reference group for clinical outcomes. Despite patients being older and having more CHC, their initial vital signs at triage showed no significant differences between the return visit and initial visit groups after age-based stratified analysis. Furthermore, among patients with ICU admission on return visits, vital signs remained similar between the first and return visit groups. Therefore, it appears that more clinical factors besides vital signs have a greater impact on the decision to arrange ICU admission or not.

Since most of the return visits with ICU admission had a primary infectious diagnosis, we analyzed WBC and CRP level. We found that, compared to the first PED visit, CRP levels were much higher on the lab tests of return visits. Based on this finding, elevated CRP levels may be of concern for infectious progression; nevertheless, infection is not the only condition that will cause CRP to rise. Illness severity based on collected clinical factors was similar in vital sign and laboratory tests between initial PED visits and return PED visits.

The mortality rates of ICU admission in our study were similar to those found in a previous multi-hospital study, in which mortality rates were around 1.3–5.0% in different hospitals’ ICU [20]. CHC affect the length of hospital stay but not mortality or ventilator use. This finding has also been observed in previous studies on general pediatric ICU admissions, where chronic medical conditions were associated with increased LOHS [16, 21]. With increased medical complexity among patients with CHC, not only patients themselves but also family factors can affect some of the decisions made in ICU practices. In 2017, Henderson et al. pointed out that parents of children with a chronic critical illness are often experts on their child’s disease [22]. This situation shifts the typical ICU clinician-parent relationship and can affect decisions regarding patient’s disposition.

Return visits with ICU admission were not associated with a higher mortality rate or increased LOHS in this study, but were related to a greater likelihood of ventilator use (aOR = 2.117). Such an observation may be rationale since the majority of mechanical ventilation support cases were due to acute respiratory failure (78%) according to a large multicenter study performed in 2012 [23]. Therefore, increased ventilator use in ICU admission of return visit patients can be a result of disease progression. In the same study, the median time of mechanical ventilation support was reported to be 5 days (interquartile range 2–8) with the mean length of the ICU stay around 10 days, which means that the few patients with respiratory complications probably do not have much impact on LOHS. Although ICU admission of PED return visits did not correlate with increased mortality or LOHS but is likely due to disease progression, caregivers may be very frustrated and disappointed if a critical condition ensues after a return PED visit. Further research should address the doctor-patient relationship and medical resource costs of ICU admission following a PED return visit.

This study has several limitations. First, this retrospective study was conducted in a single medical center, which makes applying the study results to the general population difficult, even though we provided some institutional features and prevalence data of the included population. Furthermore, some of the patients may have visited different emergency departments after being discharged from the PED in this study, but, as the biggest pediatric referral center in southern Taiwan, the likelihood of this is low considering ICU admission is the target inclusion criteria in this study. Second, the population was too small to demonstrate certain risk factors associated with previously demonstrated outcomes, such as management in PED and time of ICU admission [24, 25]. This issue may require further cooperation from multiple centers in the future for a comprehensive study. Nevertheless, this study still depicts the clinical features, outcomes, and prognostic factors of pediatric patients with ICU admission following a PED return visit.

## Conclusion

Children admitted to the ICU following an unscheduled PED return visit were rare, and most of them were ultimately discharged smoothly. Compared to those who were admitted to the ICU on an initial PED visit, patients with a return visit appeared to be older and to have more CHC. Clinical characteristics including vital signs at triage and laboratory tests showed no statistical differences between these two groups. Regarding clinical outcomes, patients admitted to the ICU on return visits were associated with higher odds of ventilator use but no differences in mortality or LOHS.

## Data Availability

The data that support the findings of this study are available from Chang Gung Memorial Hospital but restrictions apply to the availability of these data, which were used under license for the current study, and so are not publicly available. Data are however available from the authors upon reasonable request and with permission of Chang Gung Memorial Hospital.

## Abbreviations

CHC: Chronic health condition
CRP: C-reactive protein
ED: Emergency Department
ICU: Intensive Care Unit
LOHS: Length of Hospital stay
PED: Pediatric Emergency Department
WBC: White blood cell
